# Identification of Tight-Binding Plasmepsin II and Falcipain 2 Inhibitors in Aqueous Extracts of Marine Invertebrates by the Combination of Enzymatic and Interaction-Based Assays

**DOI:** 10.3390/md15040123

**Published:** 2017-04-21

**Authors:** Emir Salas-Sarduy, Yasel Guerra, Giovanni Covaleda Cortés, Francesc Xavier Avilés, María A. Chávez Planes

**Affiliations:** 1 Centro de Estudio de Proteínas, 25 # 455 entre J e I. Facultad de Biología, Universidad de la Habana, 10400 La Habana, Cuba; emirsalas@gmail.com (E.S.-S.); yaselg@gmail.com (Y.G.); 2 Coordinación Red CYTED-PROMAL (210RT0398), Proteómica y Quimiogenómica de Inhibidores de Proteasas de Origen Natural con Potencial Terapéutico en Malaria, Universidad Nacional de la Plata, 1900 La Plata, Argentina; 3 Institut de Biotecnologia i de Biomedicina and Departament de Bioquímica i de Biologia Molecular, Universitat Autònoma de Barcelona, 08193 Bellaterra (Barcelona), Spain; gcortes12@gmail.com

**Keywords:** tight-binding protease inhibitor, combined screening strategy, Plasmepsin II, Falcipain 2, *Plasmodium falciparum*

## Abstract

Natural products from marine origin constitute a very promising and underexplored source of interesting compounds for modern biotechnological and pharmaceutical industries. However, their evaluation is quite challenging and requires specifically designed assays to reliably identify the compounds of interest in a highly heterogeneous and interfering context. In the present study, we describe a general strategy for the confident identification of tight-binding protease inhibitors in the aqueous extracts of 62 Cuban marine invertebrates, using *Plasmodium falciparum* hemoglobinases Plasmepsin II and Falcipain 2 as model enzymes. To this end, we first developed a screening strategy that combined enzymatic with interaction-based assays and then validated screening conditions using five reference extracts. Interferences were evaluated and minimized. The results from the massive screening of such extracts, the validation of several hits by a variety of interaction-based assays and the purification and functional characterization of PhPI, a multifunctional and reversible tight-binding inhibitor for Plasmepsin II and Falcipain 2 from the gorgonian *Plexaura homomalla*, are presented.

## 1. Introduction

Proteases are enzymes that catalyze the hydrolysis of peptide bond in proteins and peptides [1]. Given that protein functions are dynamic in nature, they eventually undergo proteolytic processing at some point for maturation, inactivation or turnover purposes [1,2]. This fact confers to proteases an exceptional importance as functional class, provided that proteins control many aspects of cell life. Thus, proteases are involved in the control of a large number of key physiological processes, such as cell-cycle progression, cell proliferation and cell death, DNA replication, tissue remodeling, cell nutrition and signaling, immune/defensive response and pathogens invasion, among others [2,3]. Due to its high catalytic efficiency in hydrolyzing protein substrates, protease activity must be tightly regulated in vivo to avoid unwanted, unspecific or uncontrolled detrimental proteolysis. Thus, protease inhibitors, their natural counterparts, are also key physiological actors involved in a wide variety of normal, pathological and infectious processes by modulating the activity of specific protease targets [4]. Consequently, protease inhibitors have become irreplaceable molecules not only as biochemical tools for the basic study of proteases, but also as drugs for the treatment of numerous chronic and infectious human diseases [2]. In addition, protease inhibitors have found applications in many other fields, including biotechnology, agriculture and food industries [5].

During its intraerythrocytic residence, *Plasmodium falciparum*, the most virulent human malaria species, degrades massive amounts of host hemoglobin [6]. Hemoglobin catabolism occurs within an acidic organelle called digestive vacuole and is considered a critical process for *P. falciparum* survival [7]. This represents a complex proteolytic cascade performed by multiple proteases (both, exo- and endopeptidases) of different mechanistic classes (including cysteine, aspartic, and metallo proteases), which act coordinately and cooperatively to hydrolyze hemoglobin to amino acids [7,8]. Among the active aspartic hemoglobinases identified in *P. falaciparum*, Plasmepsin II (Plm II) has been the most important and best characterized. It belongs to Clan AA family A1 and shows all the typical structural and functional characteristics of pepsin-like enzymes [9,10,11], thus constituting a valuable model for this important group. Similarly, three cysteine hemoglobinases called Falcipains (FP2, FP2′ and FP3) occur within *P. falciparum* digestive vacuole. FP2 (gene ID PF11_0165) is the most abundant and best characterized, showing all the structural and functional properties of archetypical papain-like cysteine peptidases (Clan CA family C1) [12]. In addition to hemoglobin digestion, FP2 is involved in the proteolytic activation of pro-plasmepsins [13] and the release of parasites from red blood cells by degrading erythrocyte membrane skeletal proteins, including ankyrin and the band 4.1 protein [14,15]. Given its direct implication in critical parasite processes, Plm II and FP2 were considered for many years as promising chemotherapeutic targets and numerous tight-binding inhibitors classes were developed for both enzymes [16,17,18,19,20]. However, knockout parasite studies have probed both enzyme activities as redundant and/or non-essential for parasite survival in different contexts and parasite developmental stages [21,22,23], indicating that active Plm II and FP2 inhibitors reducing *P. falciparum* viability were likely operating through other (truly essential) targets and/or mechanisms of action. Despite this fact, a considerable amount of biochemical knowledge and research tools were generated around both enzymes during the last two decades. These include: efficient recombinant expression systems [24,25], crystallographic structures bound to different ligands [26,27], specific substrates and inhibitors [28,29], different kinds of High-Throughput Screening enzymatic assays [30,31,32], computational models for the virtual screening of compounds [28,33] and biophysical techniques for their characterization. This makes Plm II and FP2 exceptionally well characterized model enzymes for virtually any kind of scientific investigation.

Marine invertebrates constitute a vast and mainly unexplored source of bioactive molecules, from which have been isolated in the last decades novel compounds with biomedical and biotechnological interest [34,35,36]. Protease inhibitors have also been found abundantly in marine invertebrates [37], as part of mechanisms of chemical defenses against predation, niche displacement or associated with innate immune responses in these organisms [38,39]. Both peptidic and non-peptidic protease inhibitors isolated from marine invertebrates have shown unique features regarding their stability, enzyme specificity and tight-binding affinity (K_i_ ≤ 10^−7^ M) for their targets [40,41,42,43,44,45], anticipating a variety of potential applications. Given the high density and biodiversity of marine invertebrates, especially those from ecosystems of the tropical Caribbean Sea, it might be expected that aqueous extracts of Cuban marine invertebrates could be a valuable source of new tight-binding inhibitors for Plm II and FP2 with biomedical and/or biotechnological importance. Therefore, the ability to unambiguously identify those extracts containing the most promising inhibitors for both proteases is important to the research in natural products and the modern industry.

The main analytical approach for the identification of protease inhibitors in natural extracts has been the evaluation of inhibitory activity by using typical enzyme-specific activity assays [42,44,46,47] and to a lesser degree, interaction-based assays which sense directly the binding to the target enzyme. Enzymatic activity assays are inexpensive, high-throughput capable and provide direct information about the inhibitory effect of the extract components on the activity of the target enzyme [48]. However, they are prone to the generation of false positive hits due to the complex chemical composition of the extracts interfering with the assay (e.g., changes in pH or ionic strength, presence of competing substrates or enzymes, colored/fluorescent components affecting assay readout, etc.) during screening of crude extracts. In contrast, interaction-based assays, such as affinity chromatography [46,49], Surface Plasmon Resonance (SPR)-biosensors [50] or Intensity Fading (IF) Matrix Assisted Laser Desorption/Ionization Time-Of-Flight Mass Spectrometry (MALDI-TOF MS) [51,52], provide reliable information about the presence of target interactors even in the highly heterogeneous context of natural extracts. Nevertheless, they have been rarely used for the identification of protease inhibitors in these sources.

In this study, we screened aqueous extracts of Cuban marine invertebrates for tight-binding inhibitors of Plm II and FP2 as model enzymes of the aspartic (Clan AA family A1) and cysteine (Clan CA family C1) classes. To this end, we developed a comprehensive strategy that combines enzymatic activity assays (for the identification and prioritization of extracts) and different interaction-based methodologies (for the validation of positive hits and elimination of false positives). In a first stage, the screening conditions were established and validated using a small group of already-known (reference) extracts. Then, the selected conditions were used to screen 62 marine extracts belonging to different Phyla, leading to the confident identification of several extracts containing simultaneously tight-binding Plm II and FP2 inhibitors.

## 2. Results

### 2.1. Design and Validation of a Two-Round Screening Strategy Using Five Reference Extracts of Marine Organisms

**Figure 1:**
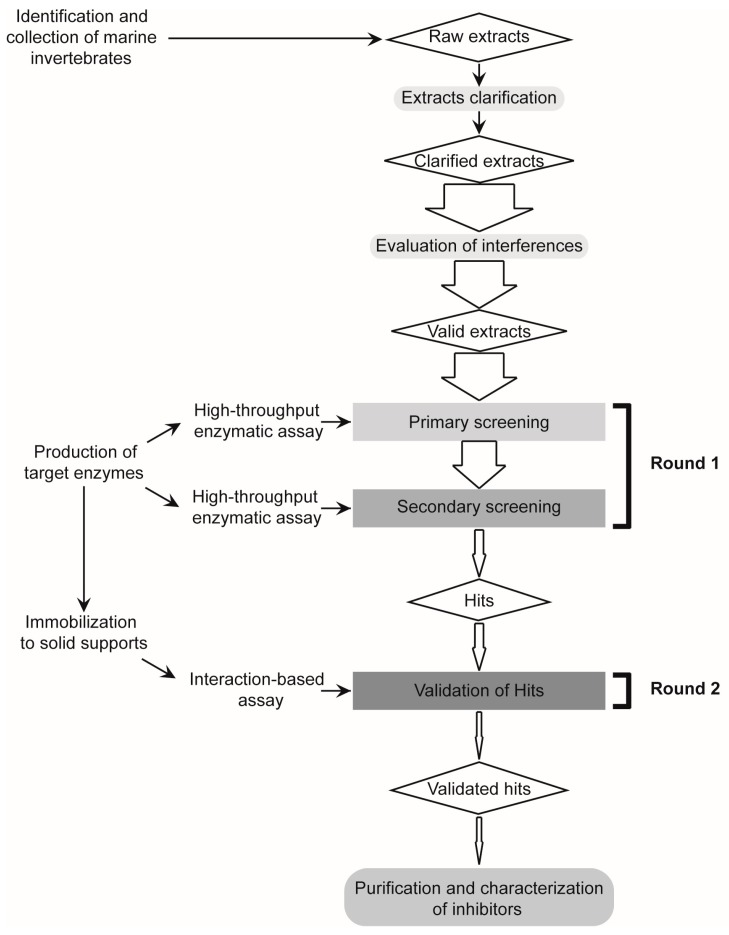
Two-round combined strategy designed for the confident identification of tight-binding protease inhibitors in complex natural extracts. Enzymatic activity assays, ordered as primary and secondary screening steps, allow the identification and prioritization of those extracts that potently inhibited the proteases of interest, whereas the interaction-based assays allow the validation of positive hits and the elimination of false positives prior to bio-guided fractionation.

To reduce the occurrence of false positive hits during the screening of tight-binding Plm II and FP2 inhibitors in natural extracts, we developed a two-round strategy combining enzymatic and interaction-based assays (Figure 1). Previous to the large-scale evaluation of more than 60 marine invertebrate extracts from Cuban coasts, we performed a validation experiment with five reference extracts, previously characterized in our lab as positives (+) or negatives (−) for inhibition against Plm II, FP2 and other related enzymes [46,47,53]: *Plexaura homomalla* (Plm II+/FP2+), *Phallusia nigra* (Plm II−/FP2−), *Stichodactyla helianthus* (Plm II+/FP2+), *Xestospongia muta* (Plm II+/FP2−) and *Polyclinum constellatum* (Plm II−/FP2−).

#### 2.1.1. Reduction of Interference Caused by Raw Extracts on Enzymatic Assays

**Figure 2:**
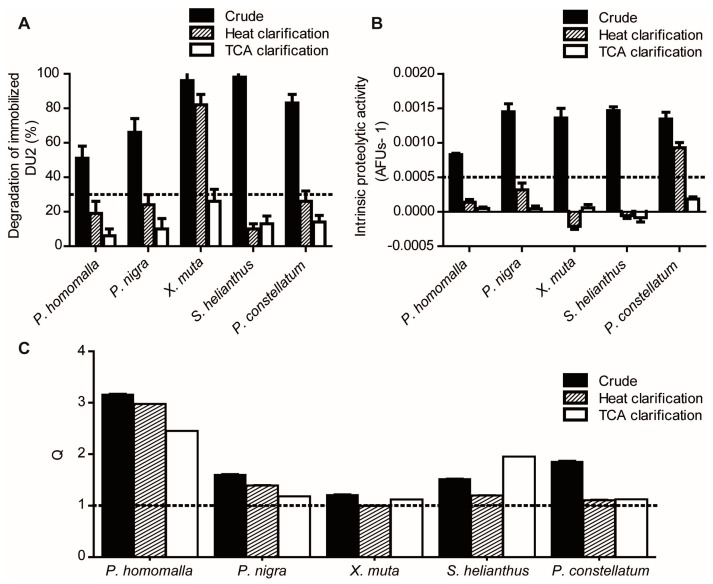
Interference levels caused by crude, heat- and TCA-clarified reference extracts on the enzymatic assays. (**A**) Intrinsic proteolytic activity of crude and clarified extracts (final dilution 1/2) on the immobilized peptidic substrate DU2 under Plm II enzymatic assay conditions (2 h at 37 °C in buffer 100 mM NaAc, pH 4.7). The proposed threshold for substrate degradation of 30% is indicated by a dashed line. (**B**) Intrinsic proteolytic activity of crude and clarified extracts (final dilution 1/20) on the fluorogenic substrate Z-FR-AMC (12.5 μM) under FP2 enzymatic assay conditions (buffer 100 mM NaAc, 10 mM DTT pH 5.5 buffer). The proposed threshold for intrinsic substrate degradation (∂F/∂t ≥ 5 × 10^−4^ AFU·s^−1^) is indicated by a dashed line. (**C**) Effects of crude and clarified extracts (final dilution 1/20) on fluorescence readouts (λexc/λemss = 355 nm/460 nm) of the AMC standard. The limit for non-significant quenching effect (*Q* = *F*_AMC_/*F*_AMC + EXT_ = 1) is indicated by a dashed line. The experiments were all performed in triplicate.

Initially, we assessed the robustness of the enzymatic assays in the presence of the crude extracts. Raw extracts caused significant substrate degradation in the absence of the target enzymes (Figure 2A,B) and in many cases, also the formation of insoluble material, thus proving unsuitable to be directly analyzed. A clarification treatment was introduced to reduce the general chemical heterogeneity of the extracts and their endogenous proteolytic activity in particular, as it may lead to the occurrence of false negatives [54]. Heat clarification (60 °C, 30 min) effectively reduced both chemical complexity and proteolytic activity to levels compatible with the assays in 80% of the extracts (Figure 2), although two heat-clarified extracts remained active against the reporter substrates, probably due to the persistence of thermo-stable proteolytic activity [47]. TCA treatment (5% final concentration) proved to be more efficacious in eliminating recalcitrant proteolytic activities (100% assay compatibility), although required further time consuming dialysis and re-concentration steps.

Previous to the evaluation with enzymatic assays, we also assessed the effect of crude, heat- and TCA-clarified reference extracts on the fluorescence readout of a standard of AMC. At the final dilution of 1/5 and 1/10, the vast majority of the extracts were able to quench the fluorescence of AMC independently of the clarification treatment (data not shown); probably due to the presence of pigments in the extracts [55]. Since this would cause an artificial decrease in the slope of enzymatic assay (mimicking the presence of a true inhibitor), it was necessary to use a final dilution (1/20) in the enzymatic assay such that the fluorescence quenching was not significant (*Q* = *F*_AMC_/*F*_AMC+EXT_ ≈ 1) (Figure 2C). Heat and TCA clarification treatments caused only a modest decrease in the observed quenching of AMC fluorescence, both reducing by 50% the number of extracts causing significant assay interferences. Even at the 1/20 dilution, *P. homomalla* heat- and TCA-clarified extracts still reduced significantly the fluorescence of AMC. In these cases, the calculated coefficient Q was used to correct the deviation of experimental assay slope.

#### 2.1.2. Screening for Tight-Binding Inhibitors Using Specific Activity Assays and Selection of Positive Hit Threshold

To favor the identification of those extracts with higher potential in terms of inhibitor potency and/or concentration, non-typical enzymatic assay conditions were adopted. First, we used relatively high concentrations of the target enzymes (0.37 μM and 0.95 μM for Plm II and FP2, respectively) for enzymatic screening. Tight-binding inhibitors display their maximal inhibitory efficiency (i.e., linear dose-response curves known as titrating behavior) when reaction conditions are such that [*E*_0_]/$K_{i}^{app}$ ≥ 10 [56]. Consequently, the extracts containing tight-binding inhibitors showing $K_{i}^{app}$ ≤ [*E*_0_]/10 (3.7 × 10^−8^ M for Plm II and 9.5 × 10^−8^ M for FP2) should display distinctive linear dose–response curves in our assays. Thus, the parameter [*E*_0_] established the threshold for inhibitor “potency” desired in the final output of the enzymatic screening. Secondly, we set the concentration of FP2 substrate around the *K_M_* value, as it equalizes the chances to identify competitive, non-competitive and uncompetitive inhibitors [57,58]. This was not necessary for Plm II assay, since E-I equilibrium is not affected by the immobilized substrate DU2 [31,59].

**Figure 3:**
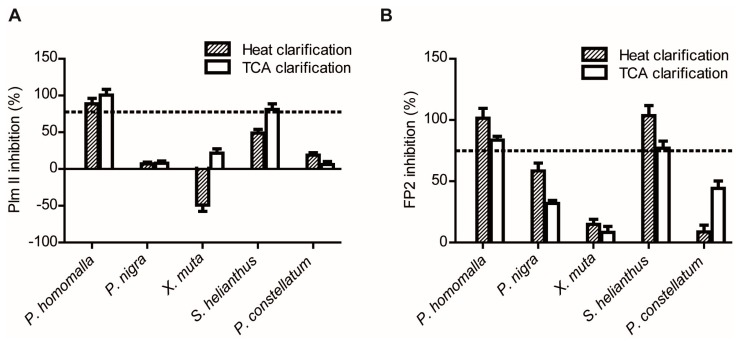
Enzymatic screening of Plm II and FP2 inhibitory activity in the reference extracts. Effect of TCA- and heat-clarified extracts on the activity of: Plm II (**A**); and FP2 (**B**) (primary screening). For both assays, extracts causing reduction levels equal or higher to 75% were considered hits. Hit thresholds are indicated by dashed lines. The experiments were all performed in triplicate.

These screening settings were subjected to validation by using the previously characterized reference extracts (Figure 3). Different levels of inhibitory activity were obtained for all clarified extracts against both enzymes. As expected, the clarified extracts of *P. homomalla* and *S. helianthus* showed high inhibitory activities against both enzymes, whereas *P. nigra* and *P. constellatum* showed only marginal inhibition. In the case of *X. muta*, we expected to find significant Plm II inhibitory activity in the heat-clarified extract [46]. Instead, a negative inhibition (which means increased substrate degradation in comparison with assay control) was observed. The intrinsic proteolytic activity present in the extract (Figure 2A) masked the identification of target-specific inhibitory activity, representing a false negative case. Thus, we established a threshold for intrinsic proteolytic activity in clarified extracts to be less than 30%–35% of the activity of control enzyme.

In addition, we established a rather high hit threshold: reduction of 75% of the enzymatic activity of the control, as it allowed both the identification of the seven most active extracts from the eight positive extracts included and the rejection of all negative and marginally inhibitory extracts (Figure 3). It is intuitively expected that the more stringent the selection criterion used, the lower the number of hits detected and concomitantly, the lower the probability of finding false positives. On the contrary, if a less stringent selection criterion is used, a higher number of hits are expected to be obtained, but also with higher probability that a non-active compound might be included by chance. Therefore, this parameter sets the level for “confidence” in the output of enzymatic screening, but also the desired inhibitor “quantity” in positive extracts. Under the titrating conditions previously established in our assays, virtually all the tight-binding inhibitor present will be in the form of E-I complex. Therefore, if all the reduction in proteolytic activity of the target enzyme is actually due to inhibition, it might be expected that the global inhibitor concentration acting on target enzyme must be at least 0.75*[*E*_0_]; i.e., 0.28 μM and 0.71 μM for Plm II and FP2 assays, respectively. In this sense, the rejection of low potential extracts is compensated by the significant reduction in false positives rates.

#### 2.1.3. Confirmatory Interaction Assays Using Affinity Chromatography, SPR and IF MALDI-TOF MS

**Figure 4:**
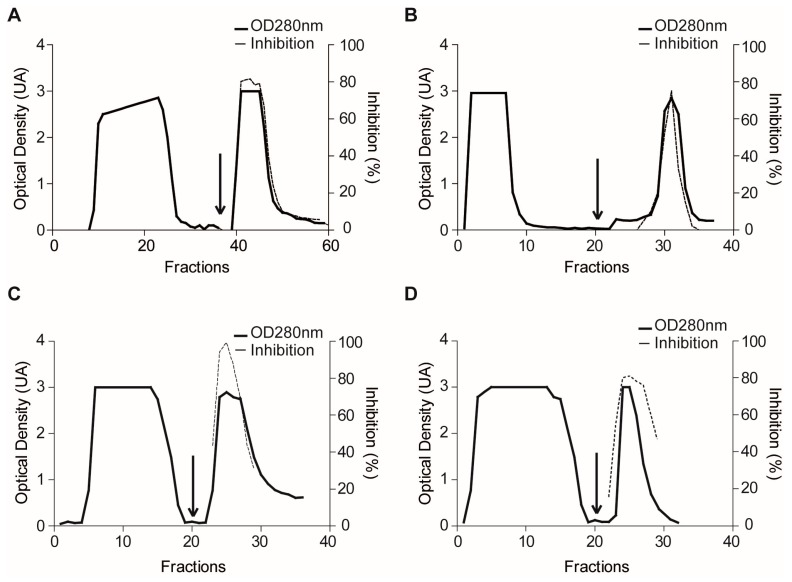
Evaluation of reference (positive) extracts for the identification of target inhibitors by using affinity chromatography. Affinity chromatography profiles of the TCA-clarified extract of: *P. homomalla* (**A**); and the heat-clarified extract of *X. muta* (**B**) with HiTrap™ Plm II-Sepharose HP resin. Affinity chromatography profiles of the heat-clarified extracts of: *P. homomalla* (**C**); and *S. helianthus* (**D**) with Papain-Sepharose resin. Arrows indicate the addition of elution buffer.

Both positive and negative extracts were then subjected to confirmatory experiments by using affinity chromatography as a reference interaction-based assay. Typical affinity profiles were obtained for the evaluation of *P. homomalla* (TCA) and *X. muta* (heat) clarified extracts on a Plm II-Sepharose matrix (Figure 4A,B), with the eluted fraction showing ~6-fold and 10-fold increase in the specific inhibitory activity, respectively [46]. Interaction of *P. homomalla* (heat) and *S. helianthus* (heat) positive extracts with clan CA family C1 enzymes were confirmed in the same way by the use of a Papain-Sepharose matrix (Figure 4C,D) [53]. In contrast, both affinity resins failed to concentrate specific inhibitory activity for *P. constellatum* (TCA), *X. muta* (TCA) and *P. nigra* (TCA) extracts (Figure S1, Supplementary Material), confirming the absence of tight-binding inhibitors of the target enzymes.

We gained further access to perform more sophisticated binding assays to some of those samples, such as SPR and IF MALDI-TOF MS, which are well-established and robust technologies to analyze protease-inhibitor interactions [52,60]. Optical Biosensor analyses were performed either using Biacore (Biacore AB, Uppsala, Sweden) (Plm II) or IAsys (ThermoLab-Systems, Cambridge, UK) (FP2) instruments, by directly immobilizing target proteases for the evaluation of serial dilutions of two reference extracts: *P. homomalla* (heat) as positive control and *X. muta* (TCA) as negative control.

At this point, the global concentration of tight-binding inhibitors active against Plm II and FP2 in the clarified extracts was estimated by titration with enzymes of known concentration. Given that the number, nature and features of the different inhibitor species present in the extracts were a priori unknown, this represented the only resource at hand to quantify the amount of functionally active inhibitors. Although this procedure did not allow us to quantify the concentration of individual inhibitors (protein isoforms, etc.), it showed three major advantages: (1) it allowed discriminating between tight-binding (those we intend to identify) and classical inhibitors (low biomedical potential); (2) unfolded or denatured inhibitor molecules (generated during extraction and/or clarification treatment) are not detected; and (3) the detection of functionally active tight-binding inhibitors is insensitive to their molecular properties (nature, molecular weight, elementary composition, etc.). In this regard, the term “apparent”, less stringent but practically convenient, is used for all the functional parameters (inhibitor concentration, *K_i_, k_ass_, k_diss_*, etc.) estimated under these circumstances, given that they represented a heterogeneous population instead of a purified inhibitor.

**Figure 5:**
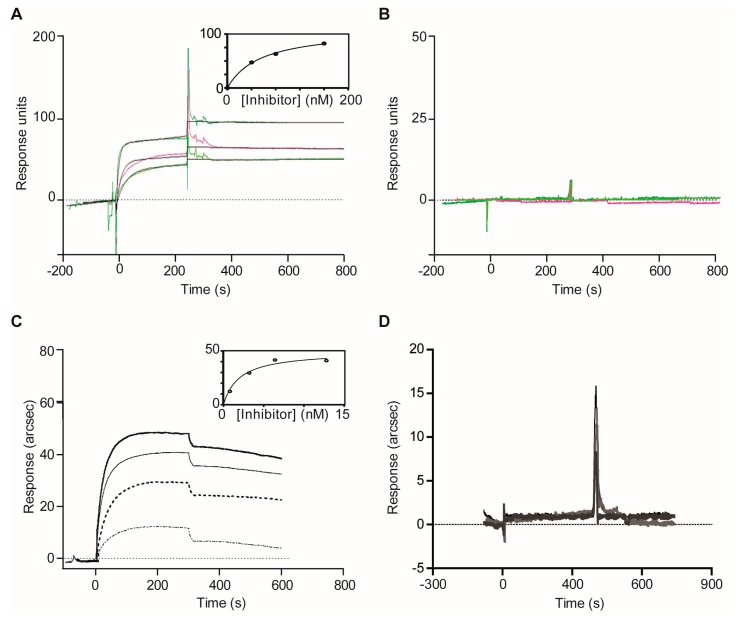
Evaluation of reference extracts for the identification of target interactors by using biosensor-based binding assays. Sensorgrams from the SPR-biosensor assay for the interaction of the heat-clarified extract of: *P. homomalla* (**A**); and the TCA-clarified extract of *X. muta* (**B**) with Plm II. Three serial (1/2) dilutions were analyzed. Sensorgrams from a resonant mirror biosensor assay for the interaction of the heat-clarified extract of: *P. homomalla* (**C**); and the TCA-clarified extract of *X. muta* (**D**) with FP2. Four dilutions were analyzed. In the case of positive interaction (**A**,**C**), insets show the steady state plots.

*P. homomalla* extract showed for both enzymes sensorgrams with typical association and dissociation phases and concentration-dependent responses indicative of actual interactions (Figure 5A,C). Further kinetic analysis of association and dissociation data allowed the determination of apparent kinetic constants *k_ass_^app^* (1.98 × 10^6^ M^−1^·s^−1^), *k_diss_^app^* (2.67 × 10^−3^ s^−1^) and *k_diss_^app^*/*k_ass_^app^* (1.35 × 10^−9^ M) for the interaction of the inhibitor with immobilized FP2, confirming the occurrence of tight-binding inhibitor in the extract [53]. Similar results were obtained for Plm II, with the interaction displaying slower association (*k_a_^app^* = 6.19 × 10^4^ M^−1^·s^−1^) and dissociation rates (*k_d_^app^* = 5.96 × 10^−3^ s^−1^) than in the previous case. The value of *k_d_^app^*/*k_a_^app^* (9.64 × 10^−9^ M), equivalent to the thermodynamic dissociation constant *K_D_^app^*, also confirmed the occurrence of tight-binding inhibitor(s) in this extract. In contrast, the extract of *X. muta* (TCA) showed no or only weak signs of interaction with immobilized FP2 and Plm II, which confirmed the absence of tight-binding inhibitors of the target enzymes (Figure 5B,D). Although this technique did not provide information about the inhibitory effects of the interaction, the kinetic and thermodynamic data obtained, such as affinity, time required to achieve steady-state and stability of the E-I complex, might be of great value to recognize potential biomedical or biotechnological applications for the enclosed inhibitor(s) [60,61].

**Figure 6:**
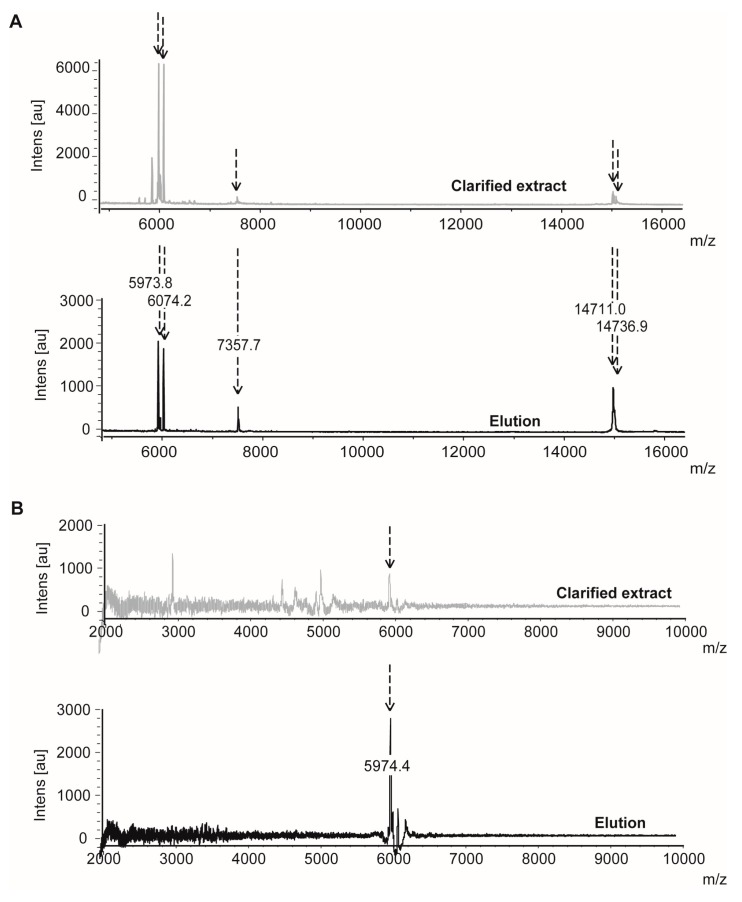
Evaluation of reference (positive) extract for the identification of target interactors by using Intensity Fading Matrix Assisted Laser Desorption/Ionization Time-Of-Flight Mass Spectrometry (IF MALDI TOF MS). (**A**) MALDI-TOF mass spectra corresponding to the heat-clarified extract of *P. homomalla* (**top**) and the elution fraction (**bottom**) after the incubation with Papain-Sepharose. (**B**) MALDI-TOF mass spectra corresponding to the heat-clarified extract of *P. homomalla* (**top**) and the elution fraction (**bottom**) after the incubation with Pepsin-Sepharose. The peaks corresponding to specific interactors are highlighted by dotted arrows.

IF MALDI-TOF MS was used as another interaction-based technique to confirm the presence of inhibitors in the selected reference extracts. Protease-inhibitor interactions are detected through the decrease (fading) of the relative intensities of the *m*/*z* signal corresponding to the inhibitor after the addition of the target protease immobilized to an appropriate support. To confirm the binding, the formed complexes are then dissociated to regenerate the faded ion signal corresponding to the inhibitor [51,52]. Analysis of the positive *P. homomalla* (heat) extract confirmed the existence of several molecules interacting specifically with Sepharose-immobilized Papain (Figure 6A), corresponding to *m*/*z*^+^ of 5973.8, 6074.2, 7357.7, 14711.0 and 14736.9 [53]. A similar analysis using Pepsin-Sepharose allowed us to identify a molecule (*m*/*z*^+^ 5974.4) specifically interacting with Pepsin-like aspartic proteases (Figure 6B), confirming in both cases the occurrence of binding partners for the targets that may be responsible for the inhibitory activity detected in the reference extract. The ion signals at *m*/*z*^+^ 5973.8 and 5974.4 detected, respectively, after specific binding analysis to Papain and Pepsin may correspond to a bi-functional (and potentially inhibitory) component in the extract, which has been previously described in marine invertebrates [40,41,44]. It is noteworthy that some ion signals were also obtained from the analysis of the negative reference *X. muta* (TCA) extract for both enzymes (data not shown). This suggests that, although able to be detected by the high sensitivity of MS, interactors of target enzymes were not inhibitory, too scant or too weak to be detected by the previous enzymatic assays. In addition to hit confirmation, this technique provided valuable information not only about the number but also the molecular weights of the potential inhibitors present in the extract, although it must be complemented with enzymatic activity assays to determine whether the interactors actually reduce activity of the target enzymes.

Noteworthy, our validation experiment using reference extracts of marine organisms are in good agreement with previous studies [46,47,53]. Although performed under different conditions, Plm II and FP2 activity assays clearly identified *S. helianthus* and *P. homomalla* as the most promising extracts against both enzymes, whereas *P. nigra* and *P. constellatum* showed the expected low potential. Their combination with binding assays further confirmed the existence of compounds specifically interacting with the target enzymes in the positive extracts and also allowed differentiation between negative and false negative hits (such as the heat-clarified extract of *X. muta* against Plm II). Taken together, our results validate not only the conditions established for individual screening assays, but also the proposed strategy; allowing its application to the large scale screening of Plm II and FP2 tight-binding inhibitors in aqueous extracts of marine invertebrates.

### 2.2. Massive Screening of Aqueous Extracts of Cuban Marine Invertebrates

**Figure 7:**
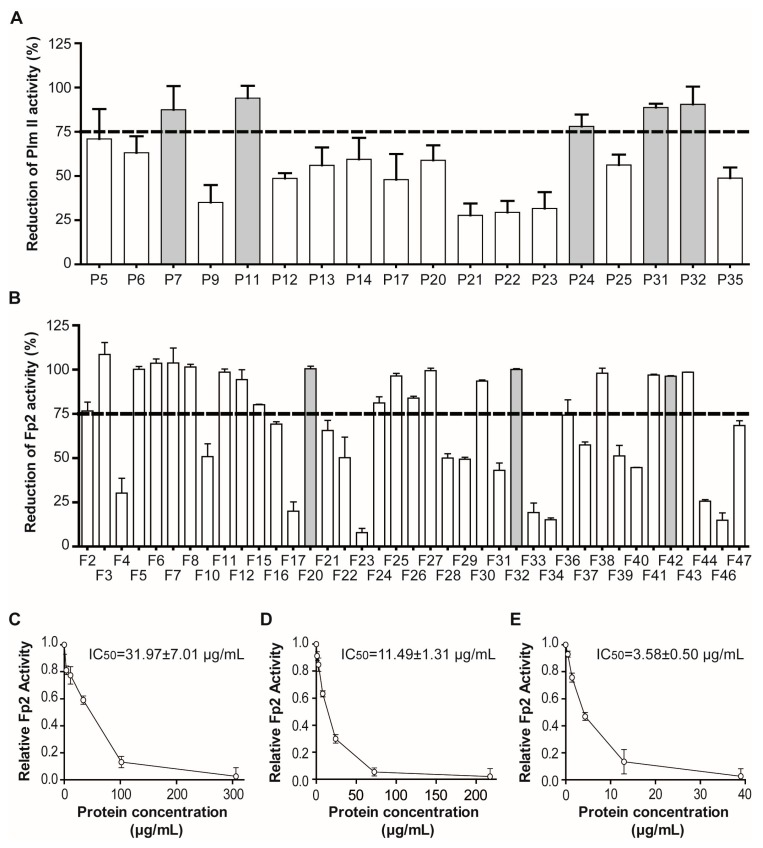
Enzymatic screening of Plm II and FP2 inhibitory activity in clarified extracts of marine invertebrates. Effect of clarified extracts on the activity of: Plm II (**A**); and FP2 (**B**) (primary screening). For both assays, extracts causing reduction levels equal or higher to 75% were considered hits. Hit thresholds are indicated by dashed lines. Biphasic dose–response curves for extracts: F20 (**C**); F32 (**D**); and F42 (**E**) selected from secondary screening against FP2. Clarified extracts selected for confirmatory experiments are indicated (solid gray). The experiments were all performed in triplicate.

A heterogeneous immuno-enzymatic Plm II assay [31] was used for the primary screening of 35 clarified aqueous extracts (Table 1), given its high-throughput compatible format. *P. homomalla* (heat), *S. helianthus* (heat and TCA), *P. nigra* (heat and TCA) and *X. muta* (heat) extracts, previously used as reference extracts to validate the strategy, were included in the screening to evaluate the consistency of our results. Seventeen clarified extracts (~49%) caused DU2 degradation levels (≥30%) incompatible with the enzymatic assay and were excluded from further evaluation (Figure S2, Supplementary Material). All the remaining extracts decreased in some level the enzymatic activity of Plm II, with values ranging from 27.7% to 94.0%. Only five extracts caused reduction levels higher than 75% and were considered positive hits (Figure 7A). Therefore, all of them (except P24 due to ambiguous initial classification and stock depletion) were considered for the confirmatory round without the need to perform a secondary screening. In addition, extracts P31 and P32 showed highly potent and reproducible Plm II inhibitory activity, in contrast to P7 and P11 that suffered inconsistencies among different isolates [46].

For FP2 tight-binding inhibitor screening, 41 clarified extracts (Table 1) were screened using a fluorogenic enzymatic assay [12]. At the established dilution (1:20), 14.6% of the clarified extracts interfered significantly with fluorescence readouts (Figure S2, Supplementary Material). For these extracts, the calculated coefficient *Q* was used to correct the experimental slope of enzymatic assays. In addition, only 2 (~5%) clarified extracts showed appreciable intrinsic proteolytic activity (∂F/∂t ≥ 5 × 10^−4^ AFU·s^−1^) against the Z-FR-AMC substrate and were excluded from further evaluation. The rest decreased in some level the enzymatic activity of FP2 control, with values ranging from 7.8% to virtually 100%. In this case, 21 extracts caused reduction levels higher than 75% and were considered positive hits (Figure 7B). Given the high rate of success (~54%), it was necessary to include a secondary screening (*IC*_50_ estimation) prior to extract validation, to establish priorities among hits. The same enzymatic assay was used in the secondary screening, as this allowed the simultaneous evaluation of several clarified extracts in the same run. Although several extracts showed biphasic dose–response curves suggesting tight-binding inhibition, three (F20, F32 and F42) displaying low *IC*_50_ values were selected for further validation (Figure 7C–E).

**Figure 8:**
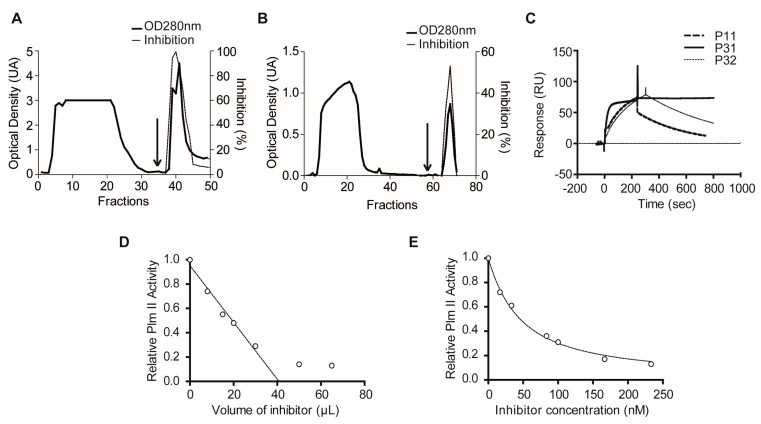
Confirmatory experiments using binding assays for molecules able to interact with Plm II in the selected extracts. Affinity chromatography profiles of extracts: P31 (**A**); and P32 (**B**) with a HiTrap™ Plm II-Sepharose HP resin. Arrows indicate the addition of elution buffer. (**C**) Sensorgrams from the SPR biosensor-based assay for the interaction of extracts P11, P31 and P32 with Plm II. (**D**) Titration curve of purified PhPI with (pepstatin A-titrated) Plm II used to determine the concentration of active inhibitor. (**E**) Concave dose–response curve for the estimation of $K_{i}^{app}$ of PhPI with Plm II.

Several non-enzymatic methodologies, able to detect specific biomolecular interactions directly, were alternatively used to confirm the existence of Plm II and FP2 inhibitors in the positive extracts selected from primary enzymatic screening. Due to the greater availability of the extracts P31 and P32, a Plm II-Sepharose affinity column was used. The typical chromatogram obtained and the increment observed in specific Plm II inhibitory activity corroborated the presence of target inhibitors in both extracts (Figure 8A,B). An SPR-based biosensor assay was used to confirm Plm II interacting molecules in the extract P11 (extracts P31 and P32 were also included for comparison) (Figure 8C). The form of the resultant sensorgrams after subtracting unspecific binding (reference cell) indicated the occurrence of Plm II binding partners in all the extracts, which are probably responsible for the inhibitory activity previously observed. In addition, it confirmed the reversibility of the interactions and indicated markedly different kinetic and thermodynamic properties for the three inhibitors. Inhibitor from P31 showed faster association and slower dissociation rates than those from P11 and P32, denoting a lower *K_i_* value for this molecule and indicating a considerably longer inhibitory effect on Plm II. Moreover, as has been previously shown, IF MALDI TOF MS analysis of P31 using Pepsin-Sepharose permitted the identification of a single Pepsin-interacting molecule with a MW of 5974 Da, and suggested that the enzyme-inhibitor interaction was reversible, which agreed with the results from SPR and affinity chromatography experiments. The Plm II inhibitor from P31 (PhPI (*Plexaura homomalla* Plasmepsin Inhibitor)) was purified to homogeneity by a combination of affinity chromatography (Plm II-Sepharose) and size exclusion chromatography (Superdex 75, Pharmacia, Sweden). The purified inhibitor was functionally characterized by using a continuous chromogenic enzymatic assay [46]. The dose–response curve of PhPI showed titrating behavior (Figure 8D), suggesting the occurrence of tight-binding inhibition and allowing us to estimate the concentration of active inhibitor. The use of lower [*E*_0_]/$K_{i}^{app}$ ratios produced a transition to concave curves (Figure 8E), which permitted the determination of a *K_i_* value of 4.3 × 10^−9^ M. This result, obtained for the purified inhibitor, is in good agreement with that previously estimated by SPR experiments (*K_D_^app^* = 9.64 × 10^−9^ M) despite the higher chemical heterogeneity of the clarified extract; indicating both the selectivity of SPR methodology and the acceptable accuracy of the estimation of global (tight-binding) inhibitor concentration by enzyme titration.

**Figure 9:**
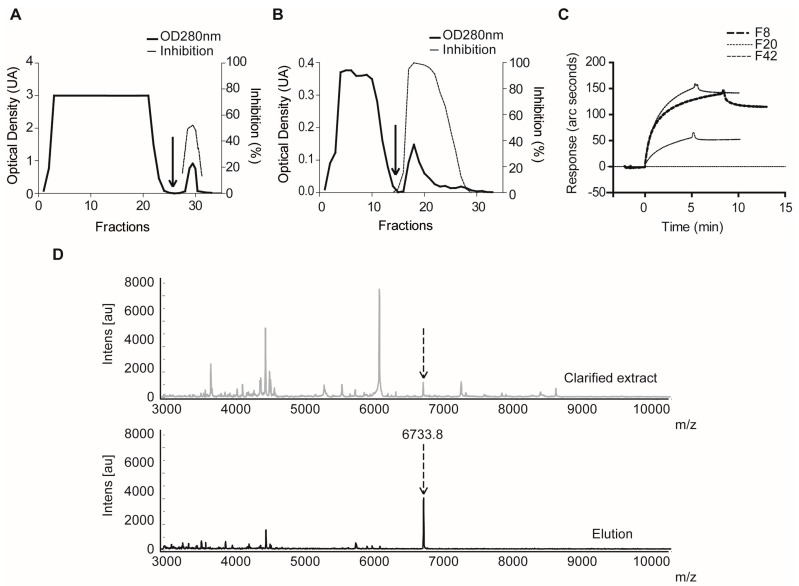
Confirmatory experiments using binding assays for molecules able to interact with Papain-like cysteine proteases in the selected extracts. Affinity chromatography profiles of extracts: F32 (**A**); and F42 (**B**) with a Papain-Sepharose resin. Arrows indicate the addition of elution buffer. (**C**) Sensorgrams from the biosensor-based assay for the interaction of extracts F20 and F42 with FP2. Reference extract F8 (positive) was included for comparison. (**D**) Matrix Assisted Laser Desorption/Ionization Time-Of-Flight (MALDI-TOF) mass spectra corresponding to the clarified extract F20 (**top**) and the elution fraction (**bottom**) after the incubation with Papain-Sepharose.

Papain-Sepharose affinity chromatography confirmed the existence of FP2 inhibitors in extracts F32 and F42, as both typical chromatograms and increased specific FP2 inhibitory activity were obtained (Figure 9A,B). A resonant-mirror optic biosensor confirmed reversible interactions for the inhibitors present in F20 and F42 with FP2, as well as similar association and dissociation behavior (Figure 9C). This behavior also proved similar to that shown by the inhibitor present in F8, which previously showed tight-binding interaction with the target enzyme and a *K_i_* value in the low nanomolar range [53]. Further information about the inhibitor present in F20 was obtained from IF MALDI TOF MS using Papain-Sepharose (Figure 9D), which showed a predominant ion signal at *m*/*z*^+^ 6733, indicative of the molecular mass for the putative inhibitor.

MALDI TOF MS provided additional information about the nature and homogeneity/heterogeneity of inhibitors here investigated, which used to be small proteins in the 5–15 kDa mass rage. This has been clearly shown in the above mentioned purified pepsin-like inhibitor from *Plexaura homomalla* (PhPI, from P31 identifier), which displayed a molecular mass of 5974 Da by such approach. A similar case was evident for the papain-like inhibitor found in *Stichodactyla helianthus* (heat clarified, F6 identifier) which showed a molecular mass of 6113 and which, after MS/MS fragmentation by either ISD and/or CID approaches, displayed derived sequences of fragments that fully matched with the internal ones of the previously reported protein inhibitor found in such species [46]. Figure S4 illustrates such case.

## 3. Discussion

The identification of tight-binding inhibitors from natural marine sources for prototypic pepsin-like and papain-like protease is of great interest, given the increasing number of applications for such bioactive molecules in biomedicine, biotechnology and industry. However, only few studies addresses the methodological complications of the identification of genuine protease inhibitors in highly heterogeneous natural extracts, a factor we presume critical for the success of screening campaigns. In this regard, this work presents a strategy for the identification and prioritization of complex natural extracts enriched in Plm II and FP2 tight binding inhibitors as a source for the isolation of potentially new bioactive compounds. The potency of the present strategy derives from the combination of medium-to-high throughput enzymatic assays with a variety of orthogonal interaction-based techniques, resulting in increased identification confidence, higher robustness to interferences and the ability to process a moderate number of natural extracts with low cost.

Both initial validation with five reference extracts and the following screening with 62 invertebrate extracts revealed some of the benefits of the selected approach. The inclusion of reference extracts in both enzymatic screenings confirmed the consistency of the results. During FP2 screening, all the reference extracts showed the same behavior as in the previous validation experiments. The same occurred during Plm II screening for the extracts of *P. homomalla* (heat)/P31, and *X. muta* (heat)/P10. Divergent results were only observed for *S. helianthus* heat- and TCA-clarified extracts. The heat-clarified extract (P3) showed high intrinsic proteolytic activity on DU2 and was excluded from screening, whereas TCA-clarified extracts (P35) inhibited ~55% of Plm II control, thus appearing negative according to our cut-off criteria. Further studies would be necessary to unravel the specific causes of these differences, although the occurrence of batch to batch variations in the balance of proteolytic/inhibitory activities is common in marine invertebrates as a consequence of biological, seasonal and environmental factors [39].

The selection of a hit threshold of 75% had a positive impact on the effectiveness of the screening as this allowed us to distinguish only those extracts with higher potential in terms of concentration and/or potency of the desired inhibitors. This was particularly important in our case, since not all the hits could be studied in parallel due to practical and economical limitations. A common definition of hit in screening assays is any activity measurement that is at least *N* (typically *N* = 3) standard deviations away from the mean of control measurements [62]. For the enzymatic assays used in this work, *N* = 3 would correspond to set hit threshold ~25% (Figure S3A). At this condition, the number of hits would be 18/18 for Plm II and 34/39 for FP2 (Figure S3B). This means that practically all the assessed extracts were able to reduce the activity of the target enzymes on the reporter substrates, statistically differing from the negative controls with a confidence >99%. However, from a biological point of view, it seems very unlikely that all of them carry specific inhibitors for the target enzymes, especially of high affinity. More probably, many of them simply might have alternative substrates for the reporter enzyme or weak inhibitors with low general interest, which probably would be eliminated during the secondary screening or the confirmation round using binding assays. In contrast, the selected 75% (which would correspond to *N* = 14.3 and *N* = 8.8 for Plm II and FP2, respectively), decreased the chance of false positives, represented a more realistic biological scenario and resulted in a better balance between positive and negative extracts to handle in practice.

Although the binding methodologies used in the study are individually sufficient to confirm the existence of target interactors, when possible, we used more than one for the most promising extracts since each technique provided different and somehow complementary information about the inhibitor or the interaction. Affinity chromatography provided direct evidence of the presence and reversibility of interacting compounds actually inhibiting the target enzymes, with the additional feature of being economically accessible to any laboratory. SPR-biosensor allowed studying not only the reversibility of the protease-ligand interaction, but also its kinetic and thermodynamic behavior. As observed in several cases along this study, the true values of kinetic and thermodynamic constants could not be determined, as inhibitor preparations were not pure. However, even under these circumstances, this technique is able to provide at least preliminary, comparative and behavioral information from the inhibitors present in different extracts, and therefore, it is useful. For instance, the time required to establish the steady state and the slope of the association phase may be related to the global rate of formation of the E-I complexes, whereas the time required for returning to the baseline is related to dissociation rates and the stability of the E-I complexes. Taken together, these elements may provide fair evidence of the existence of tight-binding interactors of the target enzyme in natural extracts, although it must be complemented with functional enzymatic evidence to confirm target inhibition.

Moreover, IF MALDI TOF MS provides information about the potential number of putative interactors, their molecular masses and their peptide/proteinaceous nature, which is critical to design appropriate purification protocols. As a consequence of the extraction with aqueous solutions and the clarification treatments introduced here, the proportion of extracted protease inhibitors is largely biased to polypeptides and proteins, in detriment of secondary metabolites (which are more likely to be extracted by mixtures of organic solvents with mid-to-low polarity). This fact complicates the determination of the detailed molecular structure for these inhibitors, as relatively large amounts of pure (i.e., recombinant) proteins are required for NMR or crystallization experiments. However, given the high sensitivity of MS, this technique may still provide valuable information regarding the primary structure of the putative inhibitor by fragmentation of the intact protein (Top-Down sequencing) or some of its peptides (Bottom-Up sequencing) [63].

Altogether, our findings highlight the aqueous extract of Cuban marine invertebrates as a prominent source for the identification of novel tight-binding (peptidic) inhibitors for pepsin-like and papain-like model proteases, such as *P. falciparum* enzymes Plm II and FP2. Interestingly, the number of hits against the cysteine hemoglobinase FP2 was significantly higher than those for the aspartyl protease Plm II, suggesting a more ubiquitous distribution of cysteine protease inhibitors in marine invertebrates. This is in good agreement with the relative abundance of both protease classes [64] and with unpublished observations of our group in previous screenings against other aspartyl and cysteine endopeptidases. Combining the results from both enzymes, the Phyla with the higher positivity ratios were *Mollusca* (4/5), *Cnidaria* (13/25) and *Porifera* (3/6) in this order, backing a role for these molecules in the regulation of endogenous proteases and/or their participation as mechanisms of chemical defenses against predation or in the innate immune response against pathogens [38,39]. A previous screening study against prototypic proteases belonging to different mechanistic classes highlighted the high frequency of protease inhibitors in Cnidarians [47]. A more comprehensive study, covering a variety of enzymes and marine species belonging to several Phyla, also showed similar results (Covaleda, Avilés and Chávez, unpublished results), confirming the validity of the data obtained here.

There are numerous examples of the medical-clinical applications of protease inhibitors [1,3], including their use in infectious diseases [65,66]. However, in most cases the small size or molecular mass and synthetic forms are favored over the high mass ones, usually protein forms, which are very frequent states for natural inhibitors. Such tendency is mainly due to the lower cost, easier obtainment, higher storage stability and lower probability of anaphylactic reactions of the former. However, the large proteinaceous protease inhibitors, quite distributed in nature, frequently display useful specificities and other biological-biomedical properties difficult to be attained by the small forms, a fact which promoted proposals and attempts for using them as drugs, diagnostic or imaging reagents. Among such cases are hirudin (from leeches), 1-antitrypsin, 2-antiplasmin and aprotinin/trasylol/BPTI (from human or mammals), other serpins, TIMPs and cystatin C (human forms), ecotin (from *E. coli*), BBI (from soya beans), etc., the first four particularly used for coagulation and fibrinolytic disorders [67]. In any case, the large natural inhibitors have been and are useful models to identify both the active site and exosite motifs of the target proteases, and are considered as lead compounds to much smaller forms by minimization, truncation and other ways to reduce the size whilst keeping the functionality [1,3].

To the moment, several aspartic and cysteine protease inhibitors have been complete or partially purified from the validated extracts [46], and others are currently under characterization. Even as enriched fractions, some of them have proven their potency as antiparasitic agents (enriched fraction of F8) against *P. falciparum* and *Trypanosoma cruzi* (the causative agents of the most deadly malaria and Chagas diseases) [53]. Further studies are needed to complete the molecular and functional characterization of such inhibitors, including complete amino-acidic sequence and selectivity toward related human proteases.

## 4. Materials and Methods

### 4.1. Collection of Marine Organisms. Preparation and Clarification of Aqueous Extracts

Marine organisms belonging to different Phyla (Table 1) were screened for inhibitory activity against Plm II and FP2. Specimens were identified “in situ” and collected by snorkeling and scuba diving from major marine habitats (seagrass beds, mangroves, coral reefs, sand bottoms and rocky coasts), from low tide to a depth of 10 m, in various locations along the northwest Cuban coast from Havana to Puerto Esperanza (Pinar del Río). All the collections were carried out during the boreal summer season (from the months of July to September).

Crude aqueous extracts were prepared by homogenizing the whole body or selected part of the wet specimen in distilled water (1:2 *w*/*v*) followed by centrifugation (10,000× *g*, 30 min, 4 °C). For clarification, the extracts were heated (60 °C, 30 min) and centrifuged (15,000× *g*, 30 min, 4 °C) to eliminate insoluble and thermolabile components. Alternatively, the crude extracts were treated with 5% final concentration of trichloroacetic acid, followed by centrifugation (15,000× *g* for 30 min at 4 °C), the supernatant was adjusted to pH 7.0 with 1 M NaOH and dialyzed (O.N., 4 °C) against distilled water (1:100 *v*/*v*) using membranes with a MW cut-off of 500 Da. The clarified extracts were kept at −20 °C until use. In all cases, total protein content was determined by the Bradford assay (Bio-Rad, Hercules, CA, USA).

### 4.2. Expression, Purification and Refolding of Plm II and FP

Plm II and FP2 were expressed as inclusion bodies in BL21(DE3)pLysS and BL21(DE3) *E. coli* strains, respectively; purified under denaturing conditions; and refolded to active enzymes as previously described [46,68].

### 4.3. Enzymatic Assays

#### 4.3.1. Plasmepsin II

A heterogeneous immunoenzymatic assay was used for the primary screening of Plm II inhibitors in clarified extracts using the biotinylated peptide DU2 (Biotin-βAβALERTF→LSFPRQSTPIGLGQALYTT-COOH) as substrate (*K_M_*: 1.6 μM; *k_cat_*: 3.5 × 10^−3^ s^−1^; *k_cat_/K_M_*: 2.2 × 10^3^ M^−1^·s^−1^; Signal sensitivity: 4.4 × 10^13^ AU∙mol^−1^) [31]. In brief, 96-well microtiter plates are coated with streptavidin. After blockade, plates are sensitized by the addition of the *N*-terminal biotinylated synthetic peptide DU2, which also comprises a Plm II cleavage sequence and a *C*-terminal epitope specifically recognized by a monoclonal antibody. After the addition of Plm II, the LERTFLSFP sequence in DU2 is recognized and hydrolyzed by the enzyme at the F–L bond, releasing the *C*-terminal epitope (RQSTPIGLGQALTYTT) which is washed away. The amount of non-degraded DU2 can then be estimated by a typical ELISA procedure, resulting in relatively low OD_492nm_ readouts when Plm II activity is high; or high OD_492nm_ readouts when Plm II is absent or effectively inhibited [31].

To evaluate the presence of interferences (e.g., proteinases that degrade DU2), clarified extracts (final dilution 1/2) were incubated for 2 h at 37 °C in 100 mM NaAc, pH 4.7 buffer with DU2-sensitized plates to evaluate their ability to degrade the immobilized substrate. Those extracts showing degradation levels equal or higher to 30% were excluded from the primary screening for inhibitors. The selected clarified extracts were pre-incubated for 10 min with Plm II (0.37 μM) at 37 °C in 100 mM NaAc pH 4.7 buffer, followed by incubation for 2 h with DU2-sensitized 96-well microtiter plates. The OD_492nm_ was recorded in a Multiskan EX plate reader (Labsystems) and data were mathematically processed as indicated by Salas et al. [31]. Extracts that caused a decrease in Plm II activity equal or higher to 75% were considered hits. All the assays were performed in triplicate. For the kinetic characterization of Plm II inhibitors, a continuous enzymatic assay was performed using Leu-Ser-Phe(NO_2_)-Nle-Ala-Leu-OMe (215 μM) as substrate (*K_M_*: 27.4 μM; *k_cat_*: 10.4 s^−1^; *k_cat_/K_M_*: 3.8 × 10^5^ M^−1^·s^−1^; Signal sensitivity: 1.0 × 10^3^ AU∙M^−1^) [46]. For this assay, Plm II (0.119–0.207 μM) was pre-incubated with the inhibitors for 10 min at 37 °C in 100 mM NaAc pH 4.4 prior the addition of the substrate. The reactions were followed at 310 nm in an Ultrospec 4000 kinetic spectrophotometer (Pharmacia Biotech, Cambridge, UK). All the assays were performed in triplicate.

#### 4.3.2. Falcipain 2

For FP2, the primary screening, the secondary screening and the characterization of partially purified inhibitors were performed with a fluorogenic enzymatic assay described previously, using Z-FR-AMC (12.5 μM) as substrate (*K_M_*: 9.1 μM; *k_cat_*: 0.4 s^−1^; *k_cat_/K_M_*: 4.5 × 10^4^ M^−1^·s^−1^; Signal sensitivity: 1.8 × 10^5^ AFU∙M^−1^) [12]. To evaluate the interferences, clarified extracts (final dilution 1/20) were mixed with an AMC standard and its ability to modify the AMC fluorescence (λexc/λemss = 355 nm/460 nm) was monitored. For those samples with *Q* = *F*_AMC_/*F*_AMC+EXT_ ≠ 1, the calculated coefficient *Q* was used to correct the value of experimental slope. The ability of clarified extracts (final dilution 1/20) to hydrolyze Z-FR-AMC (12.5 μM) was evaluated in 100 mM NaAc, 10 mM DTT pH 5.5 buffer. Those extracts showing slopes equal or higher to 5 × 10^−4^ AFU·s^−1^ were excluded from the screening.

Primary screening: Selected clarified extracts were initially pre-incubated 10 min at 37 °C with FP2 (0.95 μM) in 100 mM NaAc, 10 mM DTT pH 5.5 buffer and Z-FR-AMC (12.5 μM) was added to start reaction. Slopes in the linear region of progression curves were calculated and transformed into percentages of reduction in FP2 activity. Those extracts showing reduction levels equal or higher to 75% were considered positive hits and evaluated in a secondary screening.

Secondary Screening: All the assays (final volume 200 μL) were carried out in Greiner (black) 96-well microtiter plates and the fluorescence was recorded in a DTX 880 Multimode Reader (Beckman Coulter, Fullerton, CA, USA), except for the estimation of *K_i_*, which was carried out by using a single-cuvette AMINCO-BOWMAN SERIES 2 luminescence spectrofluorometer (Thermo Spectronic, Madison, WI, USA). Inhibition for at least 6 different inhibitor concentrations was detected through the decrease in residual activity (*a*), which is calculated by Equation (1):

(1) $$a=v_{i}/v_{0}$$

where *v_i_* and *v*_0_ are the initial rates of the reaction in the presence and absence of the inhibitor, respectively. The *IC*_50_ values (concentration of the inhibitor required to inhibit the enzyme by 50%) were estimated by fitting the Equation (2) [57] to experimental data from dose–response curves by using the GraphPad Prism program (version 5.03).

(2) $$v_{i}/v_{0}=1/\left(1+[I]/IC_{50}\right)$$

### 4.4. Estimation of the Active Concentration of Inhibitors by Titration with Enzymes

The continuous assays described for Plm II and FP2 were used to estimate the active concentration of the inhibitors. The enzymes Plm II and FP2 were previously titrated with Pepstatin A and E-64, respectively, under experimental conditions ensuring titrant behavior ([*E*_0_]/$K_{i}^{app}$ ≥ 10). Under these conditions the dose–response curve shows a biphasic behavior typical of tight-binding inhibitors. Residual enzymatic activity (*a*, Equation (1)) was evaluated after pre-incubation for 10 min of the enzyme with at least 6 different inhibitor concentrations. The titration volume was estimated by extrapolating the linear portion of curve (1> *a* >0.2) to the *x*-axis. The active concentration of the enzyme was calculated assuming a stoichiometric ratio 1:1 by using Equation (3):

(3) $${\left[E\right]}_{ACT}=[I]{}_{ACT}\cdot Vol_{ASSAY}/Vol_{TITRATION}$$

where [*E*]*_ACT_* and [*I*]*_ACT_* are the concentration of active enzyme and inhibitor, respectively, whereas *Vol_ASSAY_* is the volume of the reaction mix and *Vol_TITRATION_* is the volume of inhibitor required to completely abolish the activity of the enzyme. Titrated Plm II (0.207 μM) and FP2 (0.189 μM) were used to titrate the purified inhibitors using the same approach used to titrate the enzymes.

### 4.5. Kinetic Estimation of K_i_ Values

The continuous enzymatic assays described above were used to estimate the *K_i_* values of natural inhibitors against Plm II and FP2. Inhibition constants (*K_i_*) were determined by measuring the residual enzymatic activity (*a*) by the use of Equation (1). $K_{i}^{app}$ values were calculated by fitting the experimental data from concave dose–response curves to the Equation (4) for tight-binding inhibitors [56]:

(4) $$a=1-\frac{([E_{0}]+[I_{0}]+K_{i}^{app})-\sqrt{{\left([E_{0}]+[I_{0}]+K_{i}^{app}\right)}^{2}-4[E_{0}]\cdot [I_{0}]}}{2[E_{0}]}$$

where *a* is the residual enzymatic activity, and [*E*_0_] and [*I*_0_] are the total concentrations of enzyme and inhibitor, respectively. In all cases, the $K_{i}^{app}$ values were corrected according to the Equation (5) [56].

(5) $$K_{i}^{app}=K_{i}\cdot (1+\left[S_{0}\right]/K_{M})$$

Nonlinear regression analysis was performed by using the Statistica software for Windows (version 6.0, 2001; StatSoft, Tulsa, OK, USA).

### 4.6. Generation of the Immobilized Enzyme Support

The Glyoxyl-Sepharose derivative was prepared as described in [69]. In brief, Sepharose 4BCl was mixed by gentle stirring with ice-cold 750 mM NaBH_4_, 1.7 M NaOH solution. Glycidol was added drop-to-drop to the mixture and kept under gentle stirring for 12 h at 4 °C. After several washes with distilled water, 0.1 M NaIO_4_ was added to the Glyceryl-Sepharose gel. After 2 h of continuous mixing in darkness, the Glyoxyl-Sepharose derivative was collected by filtration, extensively washed with distilled water and conserved at 4 °C. For immobilization, Papain was coupled to Glyoxyl-Sepharose 4BCl through its amine groups at a 10 mg per-mL-of-gel ratio. The immobilization reaction was performed at RT in 100 mM sodium borate pH 10.0 buffer with gentle O.N. stirring at 4 °C. For further reduction, NaBH_4_ (14.8 mg per-mL-of-gel ratio) was added to the reaction mixture and finally, the resultant resin was washed several times with alternate cycles of pH 5.0/pH 8.8 buffers.

The immobilization of porcine Pepsin on NHS-activated Sepharose 4 Fast Flow (GE Healthcare, Buckinghamshire, UK) was performed using a procedure described previously [46]. Briefly, Pepsin was mixed with NHS-activated Sepharose at 5 mg-per-mL-of-gel ratio. Immobilization reaction was performed at RT in 20 mM Hepes pH 7.4 under gentle O.N. mixing. Ethanolamide (0.2 M, pH 8.0) was used for blocking the remaining active groups on the matrix and finally the resin was washed several times with alternate cycles of pH 4.4/8.0 buffers.

On the other hand, a preparative column for Plm II affinity chromatography was obtained by immobilization of Plm II on a pre-packed HiTrap™ NHS-Sepharose HP column (GE Healthcare, Buckinghamshire, UK) following the manufacturer’s instructions. Plm II was coupled to the matrix by applying a protein solution (8 mg total) to a 5 mL HiTrap™ NHS-Sepharose HP column at a flow rate of 0.5 mL/min. Coupling reaction was performed at RT in 0.2 M NaHCO_3_, 0.5 M NaCl pH 8.3 for 30 min. Finally, the column was washed with alternate cycles of 0.5 M ethanolamide, 0.5 M NaCl pH 8.3 and 0.1 M NaAc, 0.5 M NaCl pH 4.0 solutions.

### 4.7. Validation Experiments by Affinity Chromatography

The affinity chromatography with the HiTrap™ Plm II-Sepharose HP column (5 mL) was performed in an Äkta *purifier* system (GE Healthcare, Buckinghamshire, UK). Clarified extracts of selected hits were applied to the column previously equilibrated with 20 mM NaAc, 150 mM NaCl pH 4.4. After an exhaustive washing with the equilibration buffer to remove unspecific interactions, the bound molecules were eluted using 50 mM glycine pH 9.0 and neutralized with 1 M NaAc pH 4.4. Fractions from the different steps were collected for further Plm II inhibition analysis.

For Papain-binding analysis, clarified extracts of selected hits (10 mL) were circulated through 1 mL of a Papain-Sepharose support packed in a PD10 column and previously equilibrated with 100 mM NaAc, 150 mM NaCl, pH 5.5. After washing with sufficient volume of equilibrium buffer to reduce the OD_280nm_ to the base line, the retained components were eluted from the column with 100 mM Na_2_HPO_4_, 2 M NaCl pH 9.5 and immediately neutralized with 1 M NaAc pH 5.5. The fractions from the different steps were collected for further FP2 inhibition analysis.

### 4.8. Validation Experiments with Optical Biosensor

The analyses of the interaction of inhibitors with immobilized Plm II were performed at 25 °C on a Biacore X system (Biacore AB, Uppsala, Sweden). A 20 mM NaAc, 150 mM NaCl, 3 mM EDTA, pH 4.4 solution was used as running buffer. Purified Plm II and bovine serum albumin (BSA, used as reference) were immobilized on a carboxymethylated Sensor Chip CM5 (Biacore AB) by direct coupling to primary amines in 5 mM NaAc, pH 4.4 following the manufacturer’s instructions. For the binding experiments, a continuous flow of running buffer (50 mM NaAc, 150 mM NaCl, pH 4.4) at 10 μL/min was used, and binding responses were recorded continuously. Each 40 or 50-μL sample (final extract dilution 1/10) was injected to allow a 4–5 min association phase. After switching back to running buffer, dissociation was followed for 5–10 min. The BIAevaluation software (version 3.0, Biacore AB, Uppsala, Sweden) was used for data evaluation and estimation of apparent kinetic association (*k_a_^app^*) and dissociation (*k_d_^app^*) rate constants. The apparent parameter *k_diss_^app^*/*k_ass_^app^*, equivalent to the thermodynamic constant *K_D_^app^*, was arithmetically determined.

Kinetic analyses of interaction between FP2 and the inhibitor(s) present in pre-selected extracts were performed using an IAsys Biosensor instrument (ThermoLabSystems, Cambridge, UK). Purified FP2 was immobilized in 10 mM NaAc pH 4.0 on a carboxymethylated dextran layer (CMD cuvettes) using EDC/NHS chemistry following the manufacturer’s instructions (8–15 ng of active enzymes were immobilized in a typical experiment). For qualitative validation of pre-selected hits, extracts (final dilution 1:4000 in 50 mM NaAc, 150 mM NaCl, pH 5.5) were added to the cuvette and the association phase was recorded at 25 °C for approximately 5 min. The extract was then substituted by running buffer to collect dissociation data. For kinetic constant determination, binding curves were recorded for at least 6 different concentrations. Apparent kinetic association (*k_ass_^app^*) and dissociation (*k_diss_^app^*) rate constants were calculated separately by fitting the equations of monophasic association and dissociation models to experimental data using the FASTfit software (ThermoLabSystems). Apparent equilibrium constants (*K_D_^app^*) were determined by direct analysis of binding curves using equilibrium response data.

### 4.9. Validation Experiments by Intensity Fading MALDI-TOF MS

The experiments for the validation of selected hits were carried out by using the IF MALDI-TOF MS approach, as previously reported [52,70] using the prototypical enzymes Papain and porcine Pepsin immobilized to Sepharose 4BCl and NHS-activated Sepharose 4 Fast Flow, respectively. In the experiment, 100 μL of the pre-selected extract was mixed with 100 μL of interaction buffer 2× and 100 μL of enzyme-Sepharose suspension. After 10 min incubation at RT, the resin was collected by centrifugation and washed with 100 μL of interaction buffer (four times). Finally, the interacting molecules were eluted by changing the pH. Every fraction (sample, flow-through, every wash and eluate) was independently analyzed by MALDI-TOF MS. For Papain assay, 100 mM Na_2_HPO_4,_ 100 mM KCl, 0.1 mM EDTA, 3 mM DTT, 0.05% Brij 35, pH 6.5 was used as interaction buffer and TFA 0.1% for the elution step, whereas for Pepsin assay the interaction buffer was 50 mM NaAc, pH 4.0, and the elution of the bound molecules was carried out in successive steps using 100 μL of 0.1% TFA, 100 μL of 1% NH_4_OH and 100 μL of H_2_O:ACN:TFA (65.9:33:1), respectively.

All samples were mixed with a matrix solution (1:1, *v*/*v*) of 10 mg·mL^−1^ sinapinic acid (Bruker Daltonics, Bremen, Germany) dissolved in H_2_O:ACN:TFA (65.9:33:1); 0.5 μL of the mixture was spotted onto the MALDI-TOF groundsteel plate by using the dried-droplet method. Mass spectra were acquired in an UltrafleXtrem mass spectrometer (Bruker Daltonics, Bremen, Germany) equipped with a smartbeam™ II laser in linear-mode geometry under 20 kV and 1000 laser shots. A mixture of proteins from Bruker Daltonics (protein calibration standard I; mass range 3000–25000 Da) was used as a standard.

### 4.10. Purification of Plexaura homomalla Plasmepsin Inhibitor (PhPI) by PlmII Affinity- and Size-Exclusion Chromatography

For the affinity chromatography purification step, 5 mL of 10× equilibrium buffer (200 mM NaAc, 1.5 M NaCl pH 4.4) was added to 45 mL of the TCA-clarified extract of *P. homomalla*. The sample was loaded (linear flow rate: 12 cm/h) onto a HiTrap™ Plm II-Sepharose HP (5 mL) column, previously equilibrated with 20 mM NaAc, 150 mM NaCl pH 4.4 using an Äkta purifier system (GE Healthcare, Buckinghamshire, UK). After an exhaustive washing with the equilibrium buffer to remove unspecific interactors, the bound molecules were eluted (linear flow rate: 30 cm/h) using 50 mM glycine pH 9.0 and neutralized with 1 M NaAc pH 4.4. Fractions carrying Plm II inhibitory activity were pooled, lyophilized and solubilized in buffer 20 mM Tris-HCl, 150 mM NaCl pH 8.0. For size-exclusion chromatography purification step, the sample was loaded onto a Superdex 75 HR 10/30 column (Pharmacia, Sweden), previously equilibrated with buffer 20 mM Tris-HCl, 150 mM NaCl pH 8.0. The chromatographic run was performed on an Äkta purifier system using the same buffer at a linear flow rate of 74.6 cm/h. Fractions containing PhPI were pooled, dialyzed against abundant distilled water, lyophilized and stored at −20 °C until used.

## 5. Conclusions

As we have shown in this study, the aqueous extracts of marine invertebrates are a promising source of novel, tight-binding and peptidic/proteinaceous protease inhibitors for cysteine and, to a lesser extent, aspartic proteases (such as the *P. falciparum* hemoglobinases FP2 and Plm II), with *Cnidaria*, *Mollusca* and *Porifera* as the most prominent Phyla. Methodologically speaking, the combination of interaction-based (affinity/Surface Plasmon Resonance/and IF MALDI TOF MS) and enzymatic activity assays conveniently tuned, resulted in an efficient, powerful and versatile strategy to make a confident identification of those extracts carrying tight-binding protease inhibitors. This strategy can be easily adapted to other target enzymes and has, therefore, a high potential for improving the identification of tight-biding inhibitors from natural marine sources for a great variety of applications.

## Figures and Tables

**Table 1:** Names and Phylum distribution of the species included in this study. The identifiers (F: Falcipain 2; P: Plasmepsin II) used to distinguish each extract are indicated. The clarification treatment used in all cases was heating at 60 °C for 30 min unless otherwise indicated. Those extracts causing reduction levels equal or higher to 75% (hits) during primary screening are indicated by (+), and those further validated by interaction-based assays are indicated by (++). w.b.: whole body; s.: spikes; g.: gonads; b: body; b.c.: branchial crown; p.c.: polyps and coenenchyme; —: not tested.

Species	Phylum	Identifier	
		Falcipain 2	Plasmepsin II
*Hermodice carunculata*	*Annelida*	F30 (+)	—
*Sabellastarte magnifica* (b.c.)	*Annelida*	F22	—
*Sabellastarte magnifica* (b.)	*Annelida*	F23	—
*Bugula plumosa*	*Bryozoa*	F39	P25
*Copidozoum* sp.	*Bryozoa*	—	P24 (+)
*Zoobotrium* sp.	*Bryozoa*	—	P30
*Zoobotrium* sp. (TCA clarified)	*Bryozoa*	—	P33
*Ascidia sydneiensis*	*Chordata*	F31	—
*Ascidia sydneiensis* (TCA clarified)	*Chordata*	F44	P28
*Diplosoma listerianum*	*Chordata*	F33	—
*Ecteinascidia turbinata*	*Chordata*	F28	—
*Microcosmus* sp.	*Chordata*	—	P15
*Phallusia nigra*	*Chordata*	F21	—
*Phallusia nigra* (TCA clarified)	*Chordata*	F45	—
*Acetabularia* sp.	*Chlorophyta*	—	P17
*Bryopsis pennata*	*Chlorophyta*	—	P21
*Halimeda opuntia*	*Chlorophyta*	F34	—
*Penicillus capitatus*	*Chlorophyta*	—	P9
*Bartholomea annulata*	*Cnidaria*	—	P11 (++)
*Bunodosoma granulifera*	*Cnidaria*	F11 (+)	P8
*Bunodosoma granulifera* (TCA clarified)	*Cnidaria*	F32 (++)	—
*Cassiopeia xamachana*	*Cnidaria*	—	P29
*Condylactis gigantea*	*Cnidaria*	F3 (+)	—
*Condylactis gigantea* (TCA clarified)	*Cnidaria*	F29	—
*Eunicea calyculata*	*Cnidaria*	—	P13
*Lenubrea danae* (p.c.)	*Cnidaria*	F16	—
*Lenubrea danae* (w.b.)	*Cnidaria*	F17	—
*Lenubrea danae* (w.b.) (TCA clarified)	*Cnidaria*	F36 (+)	—
*Linuche unguiculata*	*Cnidaria*	F15 (+)	—
*Palythoa caribbaeorum*	*Cnidaria*	F2 (+)	P5
*Palythoa caribbaeorum* (TCA clarified)	*Cnidaria*	F12 (+)	—
*Physalia physalis*	*Cnidaria*	F47	—
*Plexaura homomalla*	*Cnidaria*	F8 (++)	P31 (++)
*Stichodactyla helianthus*	*Cnidaria*	F6 (++)	P3
*Stichodactyla helianthus* (TCA clarified)	*Cnidaria*	F38 (+)	P35
*Zhoanthus pulchelus*	*Cnidaria*	F7 (+)	P22
*Zhoanthus* sp.	*Cnidaria*	—	P16
*Echinaster echinophorus*	*Echinodermata*	F35	P4
*Echinometra lucunter*	*Echinodermata*	—	P23
*Eucidaris tribuloides*	*Echinodermata*	F24 (+)	—
*Holothuria mexicana*	*Echinodermata*	F42 (++)	P6
*Holothuria* sp.	*Echinodermata*	F37	—
*Holothuria* sp.	*Echinodermata*	—	P18
*Holothuria* sp.	*Echinodermata*	—	P19
*Holothuria* sp.	*Echinodermata*	—	P20
*Isostochopus badionotus*	*Echinodermata*	F26 (+)	—
*Luidia senegala*	*Echinodermata*	—	P26
*Lytechinus variegatus* (w.b.)	*Echinodermata*	F40	—
*Oreaster reticulates*	*Echinodermata*	—	P27
*Tripneustes ventricosus* (s.)	*Echinodermata*	F4	—
*Tripneustes ventricosus* (g.)	*Echinodermata*	F5 (+)	—
*Tripneustes ventricosus* (w.b.)	*Echinodermata*	F10	P2
*Cenchritis muricatus*	*Mollusca*	F43 (+)	P7 (+)
*Nerita peloronta*	*Mollusca*	F20 (++)	—
*Nerita versicolor*	*Mollusca*	F41 (+)	—
*Purpura patula*	*Mollusca*	—	P12
*Ectyoplasia ferox*	*Porifera*	—	P32 (++)
*Lissodendorix isodyctialis*	*Porifera*	F25 (+)	P1
*Xetospongia muta*	*Porifera*	F46	P10 *
*Xetospongia muta* (TCA clarified)	*Porifera*	—	P34
*Galaxaura* sp.	*Rhodophyta*	F27 (+)	—
*Laurencia* sp.	*Rhodophyta*	—	P14

* False-negative extract validated by affinity chromatography.

## Supplementary Materials

The following are available online at www.mdpi.com/1660-3397/15/4/123/s1, Figure S1: Affinity chromatography profiles of negative reference extracts used for the validation of screening strategy, Figure S2: Interference levels caused by the clarified extracts on the enzymatic assays, Figure S3: Effect of selected hit threshold on the number of hits, Figure S4: MS analysis of the affinity-eluted fraction from *S. heliant**hus* (F6 identifier) on papain-glyoxal Sepharose^®^, Figure S5: Validation of enzymatic and binding assays for FP2 and Plm II class-specific protease inhibitors.

## Abbreviations

The following abbreviations are used in this manuscript:

ACN	acetonitrile
AFU	arbitrary fluorescence units
AMC	7-amino-4-methyl coumarin
AU	absorbance units
CMD	carboxymethyl dextran
DU2	PlmII-related peptide for immunoenzymatic assays
*E. coli*	*Escherichia coli*
EDC	1-Ethyl-3-(3-dimethylaminopropyl)carbodiimide
EI	enzyme-inhibitor complex
E-64	trans-epoxysuccinyl-L-leucylamido-(4-guanidino)butane
FP2	falcipain 2
*IC*_50_	half-maximal inhibitory concentration
IF	intensity fading
MALDI-TOF	matrix assisted laser desorption/ionization time-of-flight
MS	mass spectrometry
MW	molecular weight
NaAc	sodium acetate buffer
NHS	*N*-hydroxysuccinimide
OD	Optical density
O.N.	overnight
PhPI	Plexaura homomalla plasmepsin inhibitor
Plm II	plasmepsin II
RT	room temperature
SPR	surface plasmon resonance
TCA	trichloroacetic acid
TFA	trifluoracetic acid
Z-FR-AMC	benzyloxycarbonyl-Phenyl-Arginyl-7-amino-4-methyl coumarin
