# Supplementary material for: Identification of Tight-Binding Plasmepsin II and Falcipain 2 Inhibitors in Aqueous Extracts of Marine Invertebrates by the Combination of Enzymatic and Interaction-Based Assays
Source: Mar Drugs. 2017 Apr 21;15(4):123. doi: 10.3390/md15040123 (PMC5408269; doi:10.3390/md15040123)
Supplement: Supplementary file 1 [file marinedrugs-15-00123-s001.pdf]

Electronic Supplementary Material.MarineDrugs-184239 vs2

Identification of tight-binding Plasmepsin II and Falcipain 2 inhibitors in aqueous extracts of marine invertebrates by the combination of enzymatic and interaction-based assays.

Emir Salas-Sarduy [1,3,+], Yasel Guerra [1,‡,+], Giovanni Covaleda Cortés [2], Francesc Xavier Avilés [2,3,*] and María de los Ángeles Chávez-Planes [1,3,*]

[1] Centro de Estudio de Proteínas. 25 # 455 entre J e I. Facultad de Biología, Universidad de la Habana, La Habana. Cuba; E-mails: emirsalas@gmail.com (E.S.S.); yaselg@gmail.com (Y.G); mchavez@fbio.uh.cu (M.C.P.)

[2] Institut de Biotecnologia i de Biomedicina and Departament de Bioquímica i de Biologia Molecular, Universitat Autònoma de Barcelona, 08193 Bellaterra (Barcelona), Spain; E-Mails: gcortes12@gmail.com (G.C.C.); FrancescXavier.Aviles@uab.es (F.X.A.)

[3] Red CYTED-PROMAL (210RT0398): Proteómica y Quimiogenómica de Inhibidores de Proteasas de Origen Natural con Potencial Terapéutico en Malaria. Universidad Nacional de la Plata, La Plata. Argentina.

[‡] Current address: Departamento de Medicina Molecular y Bioprocesos. Instituto de Biotecnología. Universidad Nacional Autónoma de México. Av. Universidad #2001, Col. Chamilpa CP 62210. Cuernavaca, Morelos, México.

[+] **Both authors contributed equally to this work**

*Authors to whom correspondence should be addressed; E-Mail: mchavez@fbio.uh.cu (M.C.P.); Tel.: +53 78324830. // E-Mail: FrancescXavier.Aviles@uab.es (F.X.A.); Tel.: +34 606 87 32 90.

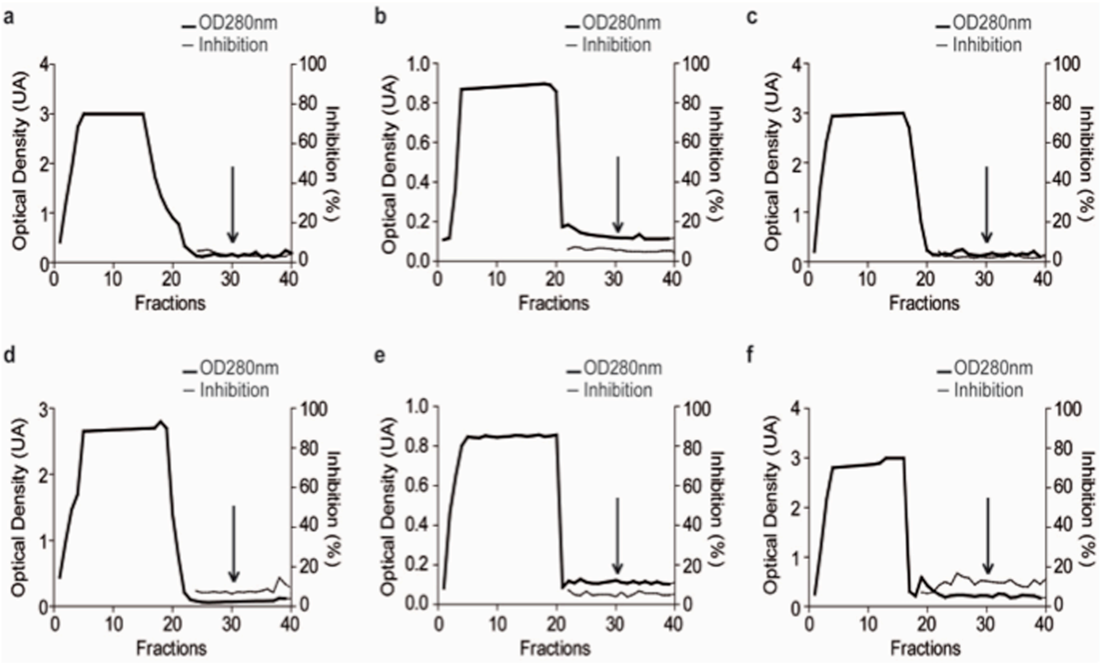

Figure S1. Affinity chromatography profiles of negative reference extracts used for the validation of screening strategy. A Plm II-Sepharose resin was used to evaluate the presence of specific inhibitors in (a) *P. nigra* (TCA), (b) *X. muta* (TCA) and (c) *P. constellatum* (TCA) extracts. A Papain-Sepharose resin was used to evaluate the presence of specific inhibitors in (d) *P. nigra* (TCA), (e) *X. muta* (TCA) and (f) *P. constellatum* (TCA) extracts. Arrows indicate the addition of elution buffer.

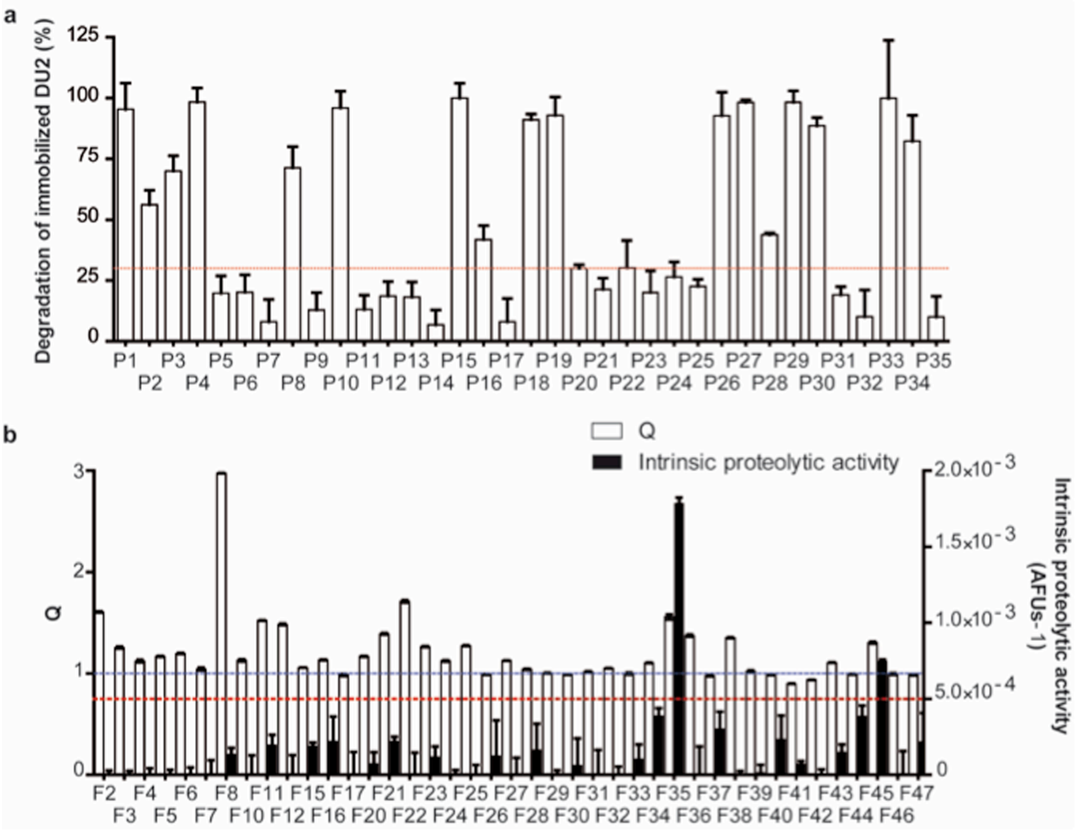

Figure S2. Interference levels caused by the clarified extracts on the enzymatic assays. (a) Intrinsic proteolytic activity of clarified extracts (final dilution 1/2) on the immobilized peptidic substrate DU2 under Plm II enzymatic assay conditions (2 h at 37 ºC in buffer 100 mM NaAc, pH 4.7). Those extracts showing degradation levels equal or higher to 30 % (----) were excluded from the screening. (b) Effects of clarified extracts (final dilution 1/20) on fluorescence readouts (λexc/λemss=355 nm/460 nm) of the AMC standard. For those extracts with $Q=F_{AMC}/F_{AMC+EXT} \neq 1$ (----), the calculated coefficient Q was used to correct the value of experimental slope in the enzymatic assay. The intrinsic proteolytic activity of clarified extracts (final dilution 1/20) on the fluorogenic substrate Z-FR-AMC (12.5 µM) was evaluated under FP2 enzymatic assay conditions (5 minutes in 100 mM NaAc, 10 mM DTT pH 5.5 buffer). Those extracts showing slopes equal or higher to $5x10^{-4}$ AFU s^{-1} (----) were excluded from the screening.

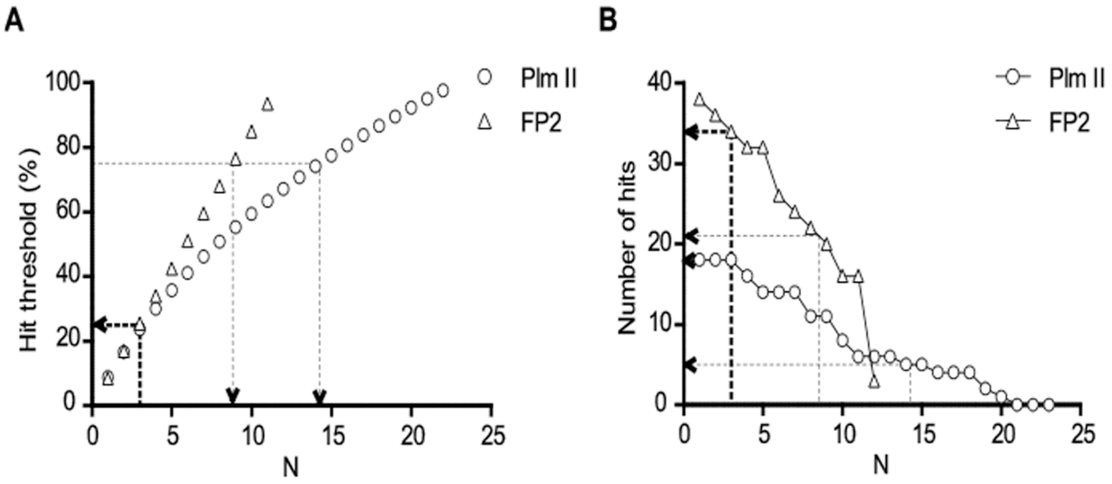

Figure S3. Effect of selected hit threshold on the number of hits. (**a**) Dependence of hit threshold with the minimum number (N) of standard deviations (SD) between hits and the means of the raw measurements of negative controls (ĉ). For Plm II assay (○), ĉ = 0.232±0.046 AU (n= 8) and for FP2 assay (Δ) ĉ = (1.416±0.12) x10-3 AFUs-1 (n=15). Hit threshold (HT) for raw measurements were calculated (HT = ĉ ± N·SD) accordingly to assay design and transformed in the equivalent percentage of reduction in enzymatic activity as previously indicated. Arrow heads indicate the calculated hit threshold (%) corresponding to N=3 and the value of N corresponding to the selected hit threshold of 75 %. (**b**) Effect of N on the number of hits for Plm II (○) and FP2 (Δ) primary screening. Arrow heads indicate the number of hits obtained for a hit threshold of 25 % (N=3) and the selected hit threshold of 75 % (N=8.8 and N=14.3 for FP2 and Plm II, respectively).

a

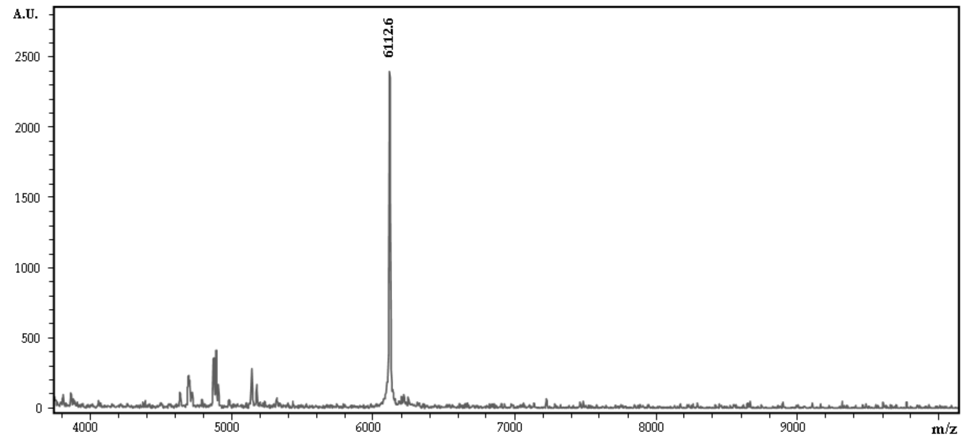

b

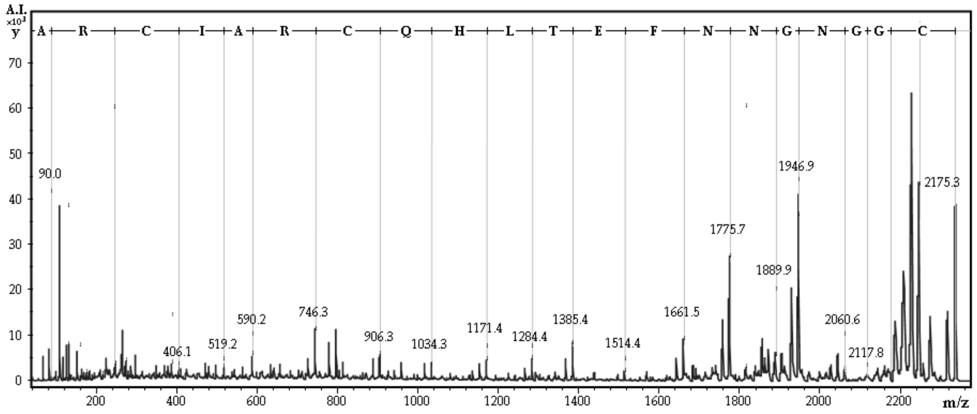

Figure S4. MS analysis of the affinity-eluted fraction from *S. helianthus* (F6 identifier) on papain-glyoxal Sepharose®. (a) visualization of the inhibitor spectral signal after the IF MALDI TOF MS procedure; (b) MALDI MS/MS (CID type) spectrum of parent m/z=2335 generated from the *S. helianthus* (F6) fraction, which allows derivation of the amino acid sequence (displayed at the top of the subfigure).

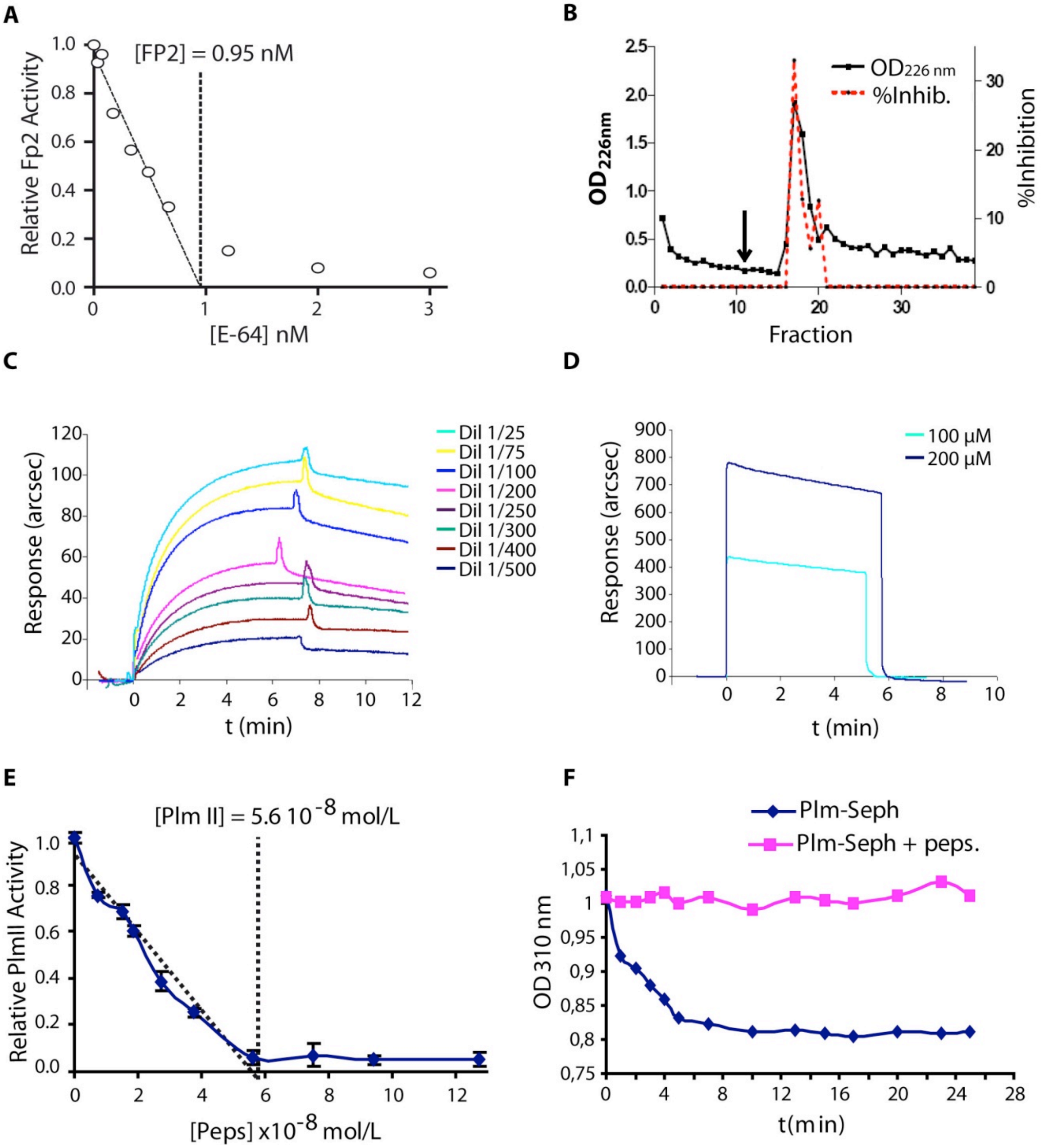

Figure S5. Validation of enzymatic and binding assays for FP2 and Plm II class-specific protease inhibitors. A) Effect of E64 on FP2 proteolytic activity using the continuous enzymatic assay previously described on Methods. This assay was used to estimate the active concentration of FP2 on the assay by titration. B) Affinity chromatography profile of the purification of natural egg white cystatin (CEW, reversible and thigh-binding inhibitor fo Clan CA family C1 enzymes) from egg whites using the

developed Papain-Sepharose resin. Addition of elution buffer is indicated by an arrow. This assay allowed to validate the functionality and specificity of the resin. C) IAsis sensorgrams correspondign to the specific interaction of immobilized FP2 with decreasing concentrations of recombinant CEW. D) Sensorgrams corresponding to a non-interacting protease inhibitor (Pepstatin A) to the same FP2 cuvette. E) Effect of Pepstatin A (reversible and thigh-binding inhibitor fo Clan AA family A1 enzymes) on Plm II proteolytic activity using the continuous enzymatic assay previously described on Methods. This assay was used to estimate the active concentration of Plm II on the assay by titration. F) Functional validation of the specificity of Plm II resin using Pepstatin A. The resin was incubated (or not) with Pepstatin A (90 nM) previous to the incubation with chromogenic substrate on activity buffer. Proteolytic activity (reduction in OD at 310 nm) was only observed for non-incubated Plm II resin, indicating the interaction of Pepstatin with the immobilized protease resulting in the abolish of its activity.
